# Mucinous Adenocarcinoma of the Colon with Clear Cell Component: A Case Report and Literature Review

**DOI:** 10.1155/2022/7631686

**Published:** 2022-12-03

**Authors:** Andrija Karačić, Gabrijela Stanić, Domagoj Štritof, Branko Bakula, Dubravka Jandrić, Inka Kekez

**Affiliations:** ^1^General Surgery Department, University Hospital Sveti Duh, Zagreb, Croatia; ^2^Pathology Department, University Hospital Sveti Duh, Zagreb, Croatia; ^3^Radiology Department, University Hospital Sveti Duh, Zagreb, Croatia

## Abstract

Clear cell adenocarcinomas of the colon are defined as a subtype of colorectal adenocarcinoma with clear cell morphology. A 65-year old man was admitted to a Gastroenterology Department for diagnostic evaluation of a tumor in the sigmoid colon found on CT. There, the patient developed complete bowel obstruction and was operated urgently, where intraoperatively, a large tumor in the sigmoid fixated to the lateral abdominal wall was revealed. A subtotal colectomy was performed. Histopathological analysis of the surgical specimen was conducted. The immunohistochemistry staining was positive for CEA, CDX2, and CD20 and negative for CK7, CD10, MUC2, AFP, and PAS staining. Mismatch repair protein testing was negative. The pathological diagnosis was mucinous carcinoma with a clear cell component which bears an extremely low incidence that has been scarcely reported in literature. This stresses the need for more case reports like ours to be published.

## 1. Background

When tumor cells display clear cytoplasms in the pathohistological analysis, the tumor is described as having clear cell morphology [1]. Carcinomas with clear cell morphology are clear cell adenocarcinoma, clear cell carcinoma, glycogen-rich adenocarcinoma, and lipid-rich adenocarcinoma [2]. Carcinomas with clear cell morphology are predominantly found not only in the kidney and Müllerian organs such as the uterus, ovaries, and Fallopian tubes but also in the salivary glands, the thyroid gland, skin, and breast. Tumor cells with clear cytoplasms are rarely observed in colorectal adenocarcinoma. Colon adenocarcinoma with a clear cell component or short clear cell adenocarcinoma of the colon (CCACC) is a well-documented subtype of colorectal cancer. But, although recognized in scientific literature, it is extremely rare. Its true incidence and prevalence are unknown, since only fewer than 30 cases have been reported in English literature [3]. In this case study, we report an additional case of clear cell colonic adenocarcinoma in a 65-year otherwise healthy man with literature review. We have put a special focus on the etiology of the clear cell tumor cells.

## 2. Case Presentation

**Figure 1:**
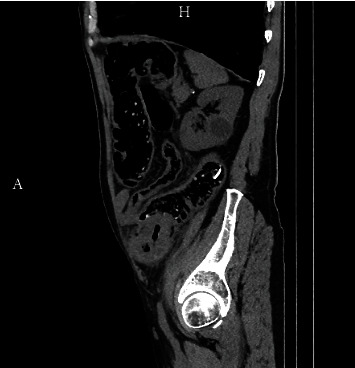
The tumor in the sigmoid fixated to the abdominal wall seen on CT in lateral cross-section.

A 65-year old man presented to the Emergency Department of a tertiary health care facility with an abdominal mass in the left lower abdomen and scarce bowel movements, nausea, vomiting, and bloating during one month. His medical history was otherwise unremarkable; he was not taking any medication. Physical examination revealed a tender abdominal mass in the left lower abdomen. His vital signs were RR 150/90 mmHg, SpO2 99%, pulse rate 83/min, and Tax 36.5°C. Beside the low hemoglobin level 115 g/l and elevated CRP level 49.5 mg/l, laboratory analysis found no abnormalities. An urgent computed tomography (CT) scan was conducted which revealed an infiltrative process in the sigmoid colon (Figure 1) and probable metastatic lesions in the liver.

The patient was admitted to Gastroenterology Department for further diagnostic work-up. At admission, tumor markers were normal, and only CA 19-9 was slightly elevated (44 kU/l). After a normal gastroscopy, a colonoscopy was planned. After laxative administration during the preparation for the procedure, the patient developed clinical and radiological signs on plain radiography of total bowel obstruction. The patient was transferred to the General Surgery Department for urgent surgery. A midline laparotomy revealed a dilated colon transversum and cecum, which was found to have a 15 cm long tear in its serosa. Further exploration revealed a tumor of the sigmoid colon the size of a male fist fixated to the lateral abdominal wall. In regard of the intra-abdominal findings, a subtotal colectomy and a terminal ileostomy with appendectomy were performed.

**Figure 2:**
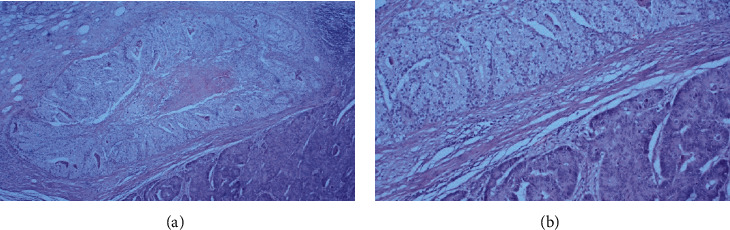
H&E slides demonstrate colonic adenocarcinoma with a clear cell component with clear cytoplasms: (a) 40×; (b) 100×.

**Figure 3:**
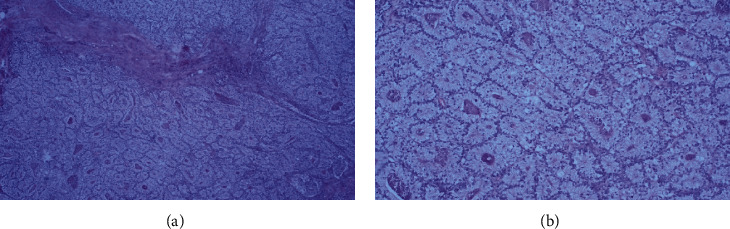
Microscopic appearance of the clear cell component in the tumor: (a) 40×; (b) 100×.

Subsequently, a thorough pathohistological analysis was conducted. The surgical specimen consisted of the resected colon and part of distal ileum. It measured 121 cm in length and contained a 10 cm long tumor. Eleven lymph nodes were isolated from the specimen with 8 of them being infiltrated by tumor metastases. Histological examination revealed mucinous colon adenocarcinoma with a clear cell component (Figures 2 and 3).

**Figure 4:**
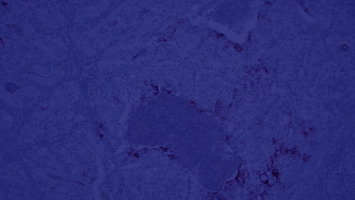
Focal distribution of CK20 positive staining in the tumor (100×).

**Figure 5:**
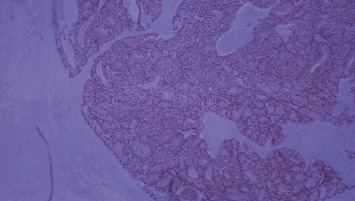
Strong nuclear CDX2 positive staining in the tumor (100×).

**Figure 6:**
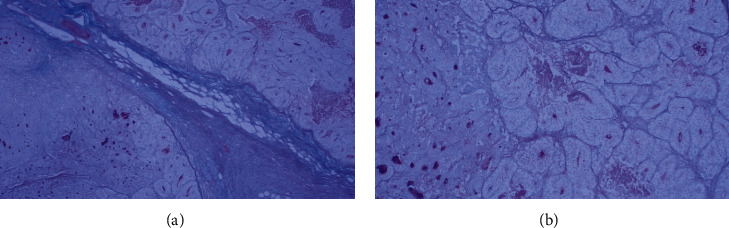
Alcian/PAS staining was negative in the tumor cells of the clear component: (a) 40×; (b) 100×.

**Figure 7:**
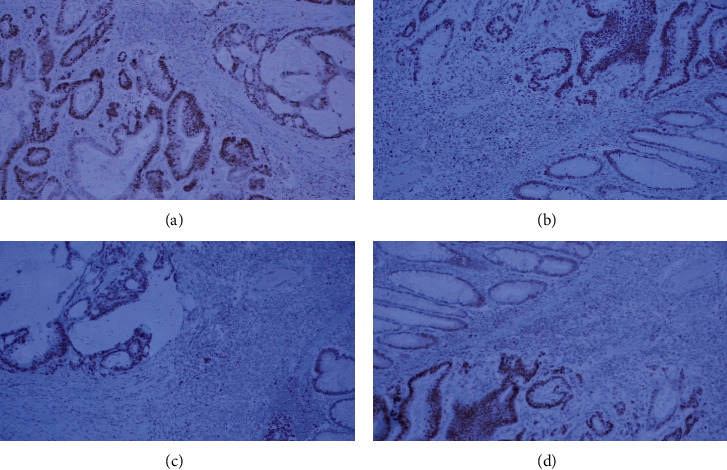
Positive staining of the clear cell component for MMR: (a) PMS2 (100×); (b) MSH6 (100×); (c) MSH2 (100×); (d) MCH1 (100×).

No concomitant adenoma in the colon specimen was found. The macroscopic and microscopic histological analysis revealed no abnormalities of the removed appendix. The differentiation of the adenocarcinoma was grade III. The tumor was breaking through the muscle layer into the adjacent adipose tissue. It did show signs of lymphovascular invasion but no signs of venous or perineural invasion. The pathological staging corresponded to pT3N2Mx. A panel of histochemical and immunohistochemical reactions was done (Figures 4–6). Mismatch repair protein (MMR) immunohistochemical testing was performed which yielded a positive reaction indicating that the tumor is negative for microsatellite instability (MSI-H) (Figure 7).

The results are summarized in Table 1.

The postoperative recovery was uneventful, and regular follow-up was normal. The postoperative diagnostic work-up, including tumor markers, endoscopy, and CT, did not detect any sign of recurrence.

## 3. Discussion

The first case of clear cell adenocarcinoma of the colon (CCACC) was reported back in 1964 by Hellstrom and Fisher. Although epidemiological information such as incidence and prevalence are not assessable, Domoto et al. retrospectively stated a probability of 0.086% for CCACC [4]. Our literature research on the PubMed database has yielded only 25 case reports of CCACC in English literature, marking our case the 26^th^ in literature. The clinical information on those 26 cases has been disclosed in Table 2.

The mean age was 58.8 years for patients with CCACC. CCACC has a predilection for males (79.2%) and the left colon (68%).

Microscopically, the clear cells in CCACC have usually pyknotic polygonal nuclei randomly arranged, not confined to the basal portion, and a clear or vacuolated cytoplasm [4]. In our case, the assessment of the nuclear typia of those clear cells was challenging, but adenocarcinoma cells accompanied them, and a transition between the two components was seen. This supported the diagnosis of the clear cell component as the atypical equivalent to the adenocarcinoma.

Differences between the conventional and clear cell component are seen in the CEA and CD10 staining results. While luminal cell apical expression of CEA is seen in well-differentiated tumors, cytoplasm expression is associated with poorly differentiated tumors [25]. CD10 expression is linked to small intestinal differentiation with higher venous invasion [26]. The joint expression of CEA in the tumor cell cytoplasm and CD10 in the clear cell component indicates its greater malignant potential.

Since it is difficult to differentiate between metastatic carcinoma, for example, metastatic clear cell carcinoma and primary CCACC if the clear cell component is dominant, confirmation of the colorectal origin of the tumor cells by immunohistochemical analysis is essential. In the pathological analysis, primary intestinal neoplasia can be differentiated by staining with cytokeratins [27–31]. Cytokeratins are composed of 20 structural proteins present in epithelial cells. Depending on the localization of the epithelial cells, the pattern of cytokeratin expression changes. CK20 is a type I keratin encoded by the gene KRT20 and has a molecular weight of 46 kDa. CK7 is a type II keratin found in nonkeratinizing epithelia and has a molecular weight of 54 kDA [16]. A positive CK20 and negative CK7 indicate intestinal origin of tumor cells with great accuracy since CK20 is expressed in the intestinal and gastric epithelial cells; whereas, CK7 is found in the breast, lung, ovary, and urothelium [28]. Along with cytokeratins, villin has also been found to be a useful support in the differential diagnosis of metastatic gastrointestinal malignancies [29]. CDX2 is another marker of intestinal neoplasia. It is a homebox transcription factor important in the development of intestinal epithelial cells. Although some authors claim that a loss of CDX2 is prevalent among dysplastic and intestinal malignant cells, newer studies identified CDX2 antibodies as a useful specific tumor marker for intestinal neoplasia [28, 29, 31]. In our case, the tumor cells stained negative for CK7 but positive for CK20 and CDX2 which indicated their colorectal origin. The histological properties of the tumor in our case are compatible with the reported cases of CCACC.

The etiology of clear cells in CCACC is still unknown. The hypothesis that the clear cell morphology is caused by glycogen accumulation [7] has been rejected by other authors [24]. Besides, glycogen, mucin and lipid accumulation, and enteroblastic differentiation are described as possible etiologies of clear cell morphology. Due to this, some cases of CCACC stain are positive for PAS [6], alcian blue [12], or AFP immunohistochemistry [19]. In our case, we could not determine the etiology of the clear cells in the tumor, but carbohydrate elution, resulting in glycogen accumulation or autolysis, and resulting in lipid accumulation are possible explanations.

Another hypothesis states that clear cell adenocarcinoma is derive from the Müllerian tubes. In literature, cases of endometriotic dysplastic glands in the colon demonstrating clear cell changes have been reported and are known as endometriosis-associated intestinal tumors (EIAT) [32]. Here again, immunohistochemical analysis can reveal the etiology.

Another theory is that carcinogenic progression from adenoma to clear cell change adenoma and eventually clear cell adenocarcinoma exists [15, 16, 24]. Due to the acute presentation in our case, this sequence could not be determined in our case.

Because of the small number of case reports, especially with adequate and long-term follow-ups, the prognosis of this subtype of colon adenocarcinoma cannot be established. But in prospect of the altered immunohistochemical profile in this subtype which is associated with greater malignant potential, one can assume that patients with CCACC have worse outcome compared to more common CRC subtypes. Four cases reported metastatic tissue either at time of diagnosis or after resection of the primary tumor [9, 19, 27, 32], which supports the hypothesis of CCACC being a potentially more malignant subtype of CRC.

Our literature review shows that, still, some major aspects of CCACC need to be further assessed. The clear cell origin in these tumors requires further elaboration. Since CCACC is so extremely rare, this is only possible through an amassing of future case reports on this pathological entity. High critical awareness among pathologists of CCACC is required because CCACC has probably more malignant behavior than other subtypes of CRC.

## Figures and Tables

**Table 1:** Summary of the tumor's immunohistochemistry report.

Marker	Result
Cytokeratin 20	Positive
Cytokeratin 10	Negative
CDX2	Positive
Cytokeratin 7	Negative
CEA	Positive
MUC2	Negative
*Α*lpha-fetoprotein (AFP)	Negative
PAS	Negative

**Table 2:** Clinicopathological information for 25 clear cell adenocarcinomas of the colon.

Author (reference)	Age	Sex	Location	Size (cm)	PAS	Alcian blue	Prognosis
Hellstrom and Fisher [5]	67	M	R	2	-/+	—	Alive
Reed et al. [6]	71	M	T	7	+	ND	ND
Jewell et al. [7]	75	M	S	0.1	—	—	Died
	56	F	S	6	ND	—	ND
Watson [8]	58	M	AC	3.5	+	—	Died
Rubio [9]	68	M	D	6	-/+	—	Died
Furman and Lauwers [10]	ND	ND	R	ND	+	ND	ND
Braumann et al. [11]	89	M	T	2.2	—	—	Died
Mallik and Katchy [12]	36	F	R	5	+	+	ND
Ko et al. [13]	62	M	S	1.5	ND	ND	ND
Hao [14]	37	M	D	12	+	—	Alive
Barisella et al. [15]	54	M	As	0.9	ND	ND	Alive
Soga et al. [16]	71	F	S	0.8	—	—	ND
Bressenot et al. [17]	84	F	D	3.5	—	—	Alive
Shi et al. [18]	52	M	R	0.9	—	ND	ND
	51	M	S	1.4	—	ND	ND
Furuya et al. [19]	81	M	As	9.5	+	ND	Died
Barrera-Maldonado et al. [3]	41	F	D	3.4	ND	ND	ND
Wang et al. [20]	26	M	T	12	ND	ND	Died
Thelin et al. [21]	25	M	As	3	ND	ND	Died
Remo et al. [22]	58	M	As	7	ND	ND	Died
	79	M	As	4.5	ND	ND	Died
Tochio et al. [23]	48	M	D	0.7	—	—	Alive
Oyama et al. [1]	58	M	S	2.5	—	—	Alive
Bakshi et al. [24]	42	M	T	4	ND	ND	ND
Current case	65	M	S	10	—	—	Alive

M: male; F: female; As: ascending colon; T: transverse colon; D: descending colon; S: sigmoid colon; R: rectum; AC: anal canal; ND: no data.

## Abbreviations

CCACC: Clear cell adenocarcinoma of the colon
CT: Computed tomography
CRP: C-reactive protein
CA 19-9: Carbohydrate antigen 19-9
CDX2: Caudal-type homebox 2
CD20: Cytokeratin 20
CK7: Cytokeratin 7
CRC: Colorectal cancer
CEA: Carcinoembryonic antigen
MMR: Mismatch repair protein
MSI: Microsatellite instability.
